# Isopropyl 3-phenyl­isoxazole-5-carboxyl­ate

**DOI:** 10.1107/S1600536813009392

**Published:** 2013-04-13

**Authors:** Li Wang, Xue-Ying Liu, Zheng-Wei Li, Sheng-Yong Zhang

**Affiliations:** a Department of Chemistry, School of Preclinical Medicine, Shanxi Medical University, 56 Xinjian South Road, 030001 Tai Yuan, People’s Republic of China; b Affiliated Hospital of Xi’an Medical College, 48 Fenggao West Road, 710077 Xi-An, People’s Republic of China; c Department of Chemistry, School of Pharmacy, Fourth Military Medical University, Changle West Road 17, 710032 Xi-An, People’s Republic of China

## Abstract

In the title compound, C_13_H_13_NO_3_, the isoxazole ring is approximately coplanar with the phenyl ring, the dihedral angle between their planes being 7.37 (19)°. In the crystal, centrosymmetrically related mol­ecules are linked into dimers by pairs of C—H⋯O hydrogen bonds, generating a ring of graph-set motif *R*_2_^2^(10).

## Related literature

For the biological activity of isoxazole derivatives, see: Angibaud *et al.* (2003 ▶). For the structure of a related compound, see: Yao & Deng (2008 ▶). For the synthesis of 3-phenyl­isoxazole-5-carb­oxy­lic acid, see: Liu *et al.* (2006 ▶).
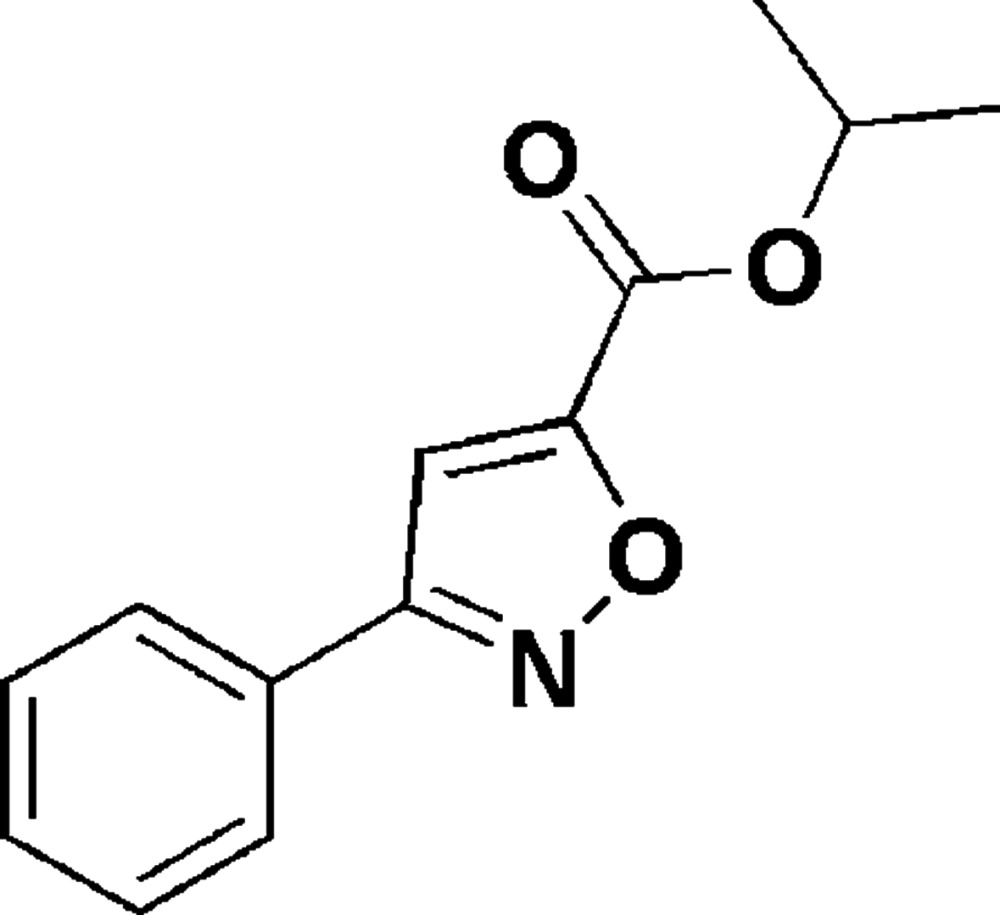


## Experimental

### 

#### Crystal data

C_13_H_13_NO_3_
*M* *_r_* = 231.24
Monoclinic, 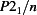

*a* = 4.6311 (10) Å
*b* = 16.596 (4) Å
*c* = 15.897 (3) Å
β = 98.321 (4)°
*V* = 1208.9 (5) Å^3^
*Z* = 4
Mo *K*α radiation
μ = 0.09 mm^−1^
*T* = 296 K
0.36 × 0.28 × 0.17 mm

#### Data collection

Bruker APEXII CCD diffractometer
Absorption correction: multi-scan (*SADABS*; Bruker, 2008 ▶) *T*_min_ = 0.968, *T*_max_ = 0.984
6039 measured reflections
2169 independent reflections
1511 reflections with *I* > 2σ(*I*)
*R*_int_ = 0.037

#### Refinement

*R*[*F* ^2^ > 2σ(*F* ^2^)] = 0.046
*wR*(*F* ^2^) = 0.126
*S* = 1.03
2169 reflections
156 parameters
H-atom parameters constrained
Δρ_max_ = 0.11 e Å^−3^
Δρ_min_ = −0.20 e Å^−3^

### 

Data collection: *APEX2* (Bruker, 2008 ▶); cell refinement: *SAINT* (Bruker, 2008 ▶); data reduction: *SAINT*; program(s) used to solve structure: *SHELXS97* (Sheldrick, 2008 ▶); program(s) used to refine structure: *SHELXL97* (Sheldrick, 2008 ▶); molecular graphics: *SHELXTL* (Sheldrick, 2008 ▶); software used to prepare material for publication: *publCIF* (Westrip, 2010 ▶).

## Supplementary Material

Crystal structure: contains datablock(s) I, global. DOI: 10.1107/S1600536813009392/rz5055sup1.cif

Structure factors: contains datablock(s) I. DOI: 10.1107/S1600536813009392/rz5055Isup2.hkl

Supplementary material file. DOI: 10.1107/S1600536813009392/rz5055Isup3.cml

Additional supplementary materials:  crystallographic information; 3D view; checkCIF report

## Figures and Tables

**Table 1:** Hydrogen-bond geometry (Å, °)

*D*—H⋯*A*	*D*—H	H⋯*A*	*D*⋯*A*	*D*—H⋯*A*
C8—H8⋯O2^i^	0.93	2.37	3.277 (2)	166

Symmetry code: (i) 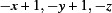.

**Fig. 1.**
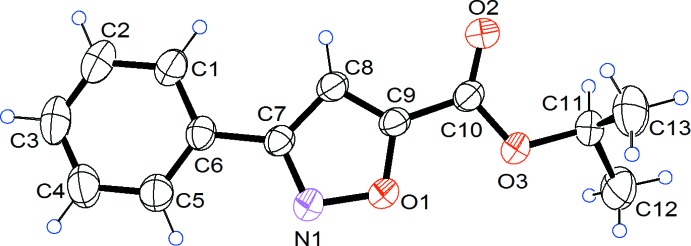
The molecular structure of the title compound, with displacement ellipsoids drawn at the 30% probability level.

**Fig. 2.**
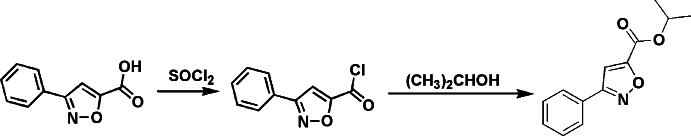
The formation of the title compound.

## supplementary crystallographic information

### Comment

Isoxazole derivatives, as useful intermediates in organic synthesis, show widespread biological activities, and are employed as antiviral drugs, antibacteria reagents, fungicide, anti-inflammatory agents, analgesics, antidepressants, anticonvulsants and pesticides (Angibaud et al., 2003). In the molecule of the title compound (Fig. 1), the dihedral angle between the phenyl and the isoxazole rings is 7.37 (19)°. The bond lengths within the isoxazole ring [C7—N1 = 1.306 (2) Å, N1—O1 = 1.402 (18) Å, O1—C9 = 1.345 (2) Å, C9—C8 = 1.327 (2) Å and C8—C7 = 1.409 (2) Å] are in agreement with those reported by Yao & Deng (2008) for 5-amino-3-(4-pyridyl)isoxazole [C7—N1 = 1.316 (18) Å, N1—O1 = 1.429 (14) Å, O1—C9 = 1.353 (17) Å, C9—C8 = 1.368 (19) Å and C8—C7 = 1.400 (19) Å]. In the crystal, centrosymmetrically related molecules are linked into dimers by C—H···O hydrogen bonds (Table 1), generating a ring of graph-set motif < i>R^2^_2_(10).

### Experimental

3-Phenylisoxazole-5-carboxylic acid (10 mmol, 1.95 g; Liu *et al.*, 2006) was dissolved in 100 ml dichloromethane, then thionyl chloride(12 mmol, 1.43 g)was dropped into the solution and stirred for 20 minutes in ice bath. The solvent was removed under reduced pressure and the mixture was used for the next step without further purification. 2-Propanol (20 mmol, 1.5 ml) was added subsequently and the mixture stirred for 6 h at room temperature. The resulting residue was purified as a white solid (1.96 g, 85% yield). Recrystallization in ethyl acetate gave fine colourless crystals suitable for X-ray study. All chemicals were purchased by Sigma Aldrich Germany.

### Refinement

All H atoms were placed in idealized positions and allowed to ride on the respective parent atom with C—H = 0.93–0.98 Å and with *U*_iso_(H) = of 1.2*U*_eq_(C) or 1.5*U*_eq_(C) for methyl H atoms.

### Crystal data

**Table:**

C_13_H_13_NO_3_	*F*(000) = 488
*M**_r_* = 231.24	*D*_x_ = 1.271 Mg m^−^^3^*D*_m_ = 1.270 Mg m^−^^3^*D*_m_ measured by not measured
Monoclinic, *P*2_1_/*n*	Mo *K*α radiation, λ = 0.71073 Å
Hall symbol: -P 2yn	Cell parameters from 1178 reflections
*a* = 4.6311 (10) Å	θ = 2.5–21.2°
*b* = 16.596 (4) Å	µ = 0.09 mm^−^^1^
*c* = 15.897 (3) Å	*T* = 296 K
β = 98.321 (4)°	Block, colourless
*V* = 1208.9 (5) Å^3^	0.36 × 0.28 × 0.17 mm
*Z* = 4	

### Data collection

**Table:**

Bruker APEXII CCD diffractometer	2169 independent reflections
Radiation source: fine-focus sealed tube	1511 reflections with *I* > 2σ(*I*)
Graphite monochromator	*R*_int_ = 0.037
φ and ω scans	θ_max_ = 25.1°, θ_min_ = 1.8°
Absorption correction: multi-scan (*SADABS*; Bruker, 2008)	*h* = −4→5
*T*_min_ = 0.968, *T*_max_ = 0.984	*k* = −19→19
6039 measured reflections	*l* = −18→17

### Refinement

**Table:**

Refinement on *F*^2^	Primary atom site location: structure-invariant direct methods
Least-squares matrix: full	Secondary atom site location: difference Fourier map
*R*[*F*^2^ > 2σ(*F*^2^)] = 0.046	Hydrogen site location: inferred from neighbouring sites
*wR*(*F*^2^) = 0.126	H-atom parameters constrained
*S* = 1.03	*w* = 1/[σ^2^(*F*_o_^2^) + (0.0611*P*)^2^ + 0.0883*P*] where *P* = (*F*_o_^2^ + 2*F*_c_^2^)/3
2169 reflections	(Δ/σ)_max_ < 0.001
156 parameters	Δρ_max_ = 0.11 e Å^−^^3^
0 restraints	Δρ_min_ = −0.20 e Å^−^^3^

### Special details

**Table:**

Geometry. All e.s.d.'s (except the e.s.d. in the dihedral angle between two l.s. planes) are estimated using the full covariance matrix. The cell e.s.d.'s are taken into account individually in the estimation of e.s.d.'s in distances, angles and torsion angles; correlations between e.s.d.'s in cell parameters are only used when they are defined by crystal symmetry. An approximate (isotropic) treatment of cell e.s.d.'s is used for estimating e.s.d.'s involving l.s. planes.
Refinement. Refinement of *F*^2^ against ALL reflections. The weighted *R*-factor *wR* and goodness of fit *S* are based on *F*^2^, conventional *R*-factors *R* are based on *F*, with *F* set to zero for negative *F*^2^. The threshold expression of *F*^2^ > σ(*F*^2^) is used only for calculating *R*-factors(gt) *etc*. and is not relevant to the choice of reflections for refinement. *R*-factors based on *F*^2^ are statistically about twice as large as those based on *F*, and *R*- factors based on ALL data will be even larger.

### Fractional atomic coordinates and isotropic or equivalent isotropic displacement parameters (Å^2^)

**Table:**

	*x*	*y*	*z*	*U*_iso_*/*U*_eq_	
N1	1.1576 (4)	0.66232 (10)	0.15016 (9)	0.0630 (5)	
O1	1.0131 (3)	0.60739 (8)	0.19660 (7)	0.0607 (4)	
O2	0.4550 (3)	0.46825 (8)	0.12822 (8)	0.0746 (5)	
O3	0.6627 (3)	0.50549 (7)	0.25802 (7)	0.0576 (4)	
C1	0.9997 (5)	0.69977 (13)	−0.07802 (12)	0.0712 (6)	
H1	0.8549	0.6615	−0.0930	0.085*	
C2	1.0757 (6)	0.75230 (15)	−0.13884 (14)	0.0828 (7)	
H2	0.9802	0.7496	−0.1944	0.099*	
C3	1.2896 (6)	0.80792 (14)	−0.11749 (17)	0.0844 (7)	
H3	1.3391	0.8433	−0.1584	0.101*	
C4	1.4315 (5)	0.81193 (13)	−0.03635 (16)	0.0845 (7)	
H4	1.5793	0.8496	−0.0222	0.101*	
C5	1.3566 (5)	0.76046 (12)	0.02457 (14)	0.0681 (6)	
H5	1.4541	0.7636	0.0799	0.082*	
C6	1.1380 (4)	0.70408 (10)	0.00457 (11)	0.0521 (5)	
C7	1.0454 (4)	0.65230 (10)	0.07063 (10)	0.0491 (4)	
C8	0.8306 (4)	0.59153 (11)	0.06221 (10)	0.0530 (5)	
H8	0.7213	0.5728	0.0124	0.064*	
C9	0.8187 (4)	0.56698 (10)	0.14107 (10)	0.0492 (5)	
C10	0.6272 (4)	0.50799 (11)	0.17418 (11)	0.0525 (5)	
C11	0.4715 (5)	0.45151 (12)	0.29747 (12)	0.0629 (5)	
H11	0.2788	0.4501	0.2627	0.075*	
C12	0.4454 (7)	0.48786 (17)	0.38172 (15)	0.1041 (10)	
H12A	0.6342	0.4896	0.4158	0.156*	
H12B	0.3159	0.4558	0.4099	0.156*	
H12C	0.3692	0.5416	0.3738	0.156*	
C13	0.6001 (6)	0.36909 (14)	0.30211 (16)	0.0981 (8)	
H13A	0.6165	0.3505	0.2458	0.147*	
H13B	0.4765	0.3331	0.3280	0.147*	
H13C	0.7901	0.3705	0.3355	0.147*	

### Atomic displacement parameters (Å^2^)

**Table:**

	*U*^11^	*U*^22^	*U*^33^	*U*^12^	*U*^13^	*U*^23^
N1	0.0788 (12)	0.0650 (10)	0.0460 (9)	−0.0149 (9)	0.0112 (8)	0.0018 (7)
O1	0.0757 (9)	0.0651 (8)	0.0408 (7)	−0.0141 (7)	0.0070 (6)	0.0003 (6)
O2	0.0940 (11)	0.0756 (9)	0.0512 (8)	−0.0266 (8)	0.0007 (8)	−0.0020 (7)
O3	0.0695 (9)	0.0626 (8)	0.0410 (7)	−0.0099 (7)	0.0085 (6)	0.0042 (6)
C1	0.0870 (16)	0.0752 (14)	0.0524 (12)	−0.0040 (12)	0.0136 (11)	0.0074 (11)
C2	0.1036 (19)	0.0902 (17)	0.0575 (13)	0.0099 (15)	0.0219 (13)	0.0172 (12)
C3	0.111 (2)	0.0674 (15)	0.0847 (18)	0.0077 (14)	0.0463 (16)	0.0227 (13)
C4	0.1026 (19)	0.0696 (15)	0.0871 (17)	−0.0156 (13)	0.0332 (15)	0.0076 (13)
C5	0.0797 (15)	0.0618 (12)	0.0650 (13)	−0.0042 (11)	0.0182 (11)	0.0010 (10)
C6	0.0606 (12)	0.0479 (10)	0.0505 (11)	0.0045 (9)	0.0171 (9)	0.0010 (8)
C7	0.0570 (11)	0.0483 (10)	0.0427 (10)	0.0027 (9)	0.0095 (8)	−0.0019 (8)
C8	0.0630 (12)	0.0541 (11)	0.0409 (10)	−0.0005 (9)	0.0043 (9)	−0.0014 (8)
C9	0.0592 (11)	0.0474 (10)	0.0402 (10)	0.0009 (9)	0.0047 (8)	−0.0036 (8)
C10	0.0662 (12)	0.0493 (10)	0.0417 (10)	0.0033 (9)	0.0068 (9)	0.0004 (8)
C11	0.0712 (13)	0.0637 (13)	0.0550 (11)	−0.0105 (10)	0.0132 (10)	0.0104 (9)
C12	0.147 (3)	0.107 (2)	0.0693 (16)	−0.0330 (18)	0.0530 (17)	−0.0100 (14)
C13	0.122 (2)	0.0687 (15)	0.107 (2)	0.0054 (15)	0.0270 (17)	0.0262 (14)

### Geometric parameters (Å, º)

**Table:**

N1—C7	1.306 (2)	C5—H5	0.9300
N1—O1	1.4022 (18)	C6—C7	1.468 (2)
O1—C9	1.345 (2)	C7—C8	1.409 (2)
O2—C10	1.198 (2)	C8—C9	1.327 (2)
O3—C10	1.3197 (19)	C8—H8	0.9300
O3—C11	1.463 (2)	C9—C10	1.469 (3)
C1—C6	1.377 (3)	C11—C13	1.489 (3)
C1—C2	1.384 (3)	C11—C12	1.490 (3)
C1—H1	0.9300	C11—H11	0.9800
C2—C3	1.361 (3)	C12—H12A	0.9600
C2—H2	0.9300	C12—H12B	0.9600
C3—C4	1.362 (3)	C12—H12C	0.9600
C3—H3	0.9300	C13—H13A	0.9600
C4—C5	1.373 (3)	C13—H13B	0.9600
C4—H4	0.9300	C13—H13C	0.9600
C5—C6	1.381 (3)		
			
C7—N1—O1	105.89 (14)	C7—C8—H8	127.6
C9—O1—N1	107.68 (12)	C8—C9—O1	110.58 (16)
C10—O3—C11	117.22 (15)	C8—C9—C10	130.77 (17)
C6—C1—C2	120.2 (2)	O1—C9—C10	118.60 (15)
C6—C1—H1	119.9	O2—C10—O3	124.97 (18)
C2—C1—H1	119.9	O2—C10—C9	122.10 (16)
C3—C2—C1	120.1 (2)	O3—C10—C9	112.92 (16)
C3—C2—H2	119.9	O3—C11—C13	108.73 (18)
C1—C2—H2	119.9	O3—C11—C12	105.69 (16)
C2—C3—C4	120.2 (2)	C13—C11—C12	114.28 (19)
C2—C3—H3	119.9	O3—C11—H11	109.3
C4—C3—H3	119.9	C13—C11—H11	109.3
C3—C4—C5	120.1 (2)	C12—C11—H11	109.3
C3—C4—H4	120.0	C11—C12—H12A	109.5
C5—C4—H4	120.0	C11—C12—H12B	109.5
C4—C5—C6	120.7 (2)	H12A—C12—H12B	109.5
C4—C5—H5	119.6	C11—C12—H12C	109.5
C6—C5—H5	119.6	H12A—C12—H12C	109.5
C1—C6—C5	118.61 (18)	H12B—C12—H12C	109.5
C1—C6—C7	120.53 (18)	C11—C13—H13A	109.5
C5—C6—C7	120.80 (17)	C11—C13—H13B	109.5
N1—C7—C8	111.06 (15)	H13A—C13—H13B	109.5
N1—C7—C6	120.10 (16)	C11—C13—H13C	109.5
C8—C7—C6	128.77 (16)	H13A—C13—H13C	109.5
C9—C8—C7	104.79 (16)	H13B—C13—H13C	109.5
C9—C8—H8	127.6		
			
C7—N1—O1—C9	−0.11 (18)	N1—C7—C8—C9	0.9 (2)
C6—C1—C2—C3	0.7 (3)	C6—C7—C8—C9	−176.01 (17)
C1—C2—C3—C4	0.4 (4)	C7—C8—C9—O1	−0.9 (2)
C2—C3—C4—C5	−0.8 (4)	C7—C8—C9—C10	176.46 (18)
C3—C4—C5—C6	0.1 (3)	N1—O1—C9—C8	0.68 (19)
C2—C1—C6—C5	−1.4 (3)	N1—O1—C9—C10	−177.06 (15)
C2—C1—C6—C7	175.88 (18)	C11—O3—C10—O2	−1.7 (3)
C4—C5—C6—C1	0.9 (3)	C11—O3—C10—C9	176.98 (15)
C4—C5—C6—C7	−176.28 (18)	C8—C9—C10—O2	5.0 (3)
O1—N1—C7—C8	−0.5 (2)	O1—C9—C10—O2	−177.82 (17)
O1—N1—C7—C6	176.73 (14)	C8—C9—C10—O3	−173.73 (18)
C1—C6—C7—N1	−173.13 (18)	O1—C9—C10—O3	3.5 (2)
C5—C6—C7—N1	4.0 (3)	C10—O3—C11—C13	84.7 (2)
C1—C6—C7—C8	3.5 (3)	C10—O3—C11—C12	−152.16 (19)
C5—C6—C7—C8	−179.33 (18)		

### Hydrogen-bond geometry (Å, º)

**Table:**

*D*—H···*A*	*D*—H	H···*A*	*D*···*A*	*D*—H···*A*
C8—H8···O2^i^	0.93	2.37	3.277 (2)	166

Symmetry code: (i) −*x*+1, −*y*+1, −*z*.
